# Evaluating the transitions in care for children presenting with acute asthma to emergency departments: a retrospective cohort study

**DOI:** 10.1186/s12873-021-00550-z

**Published:** 2021-12-07

**Authors:** Kimberly R. Kroetch, Brian H. Rowe, Rhonda J. Rosychuk

**Affiliations:** 1 Department of Mathematics and Statistics, Faculty of Arts and Science, MacEwan University, Edmonton, Alberta T5J 4S2 Canada; 2 Department of Emergency Medicine, Faculty of Medicine & Dentistry, University of Alberta, Edmonton, Alberta T6G 2R7 Canada; 3 School of Public Health, University of Alberta, Edmonton, Alberta Canada; 4 Department of Medicine, Faculty of Medicine & Dentistry, University of Alberta, Edmonton, Alberta Canada; 5 Department of Pediatrics, Faculty of Medicine & Dentistry, University of Alberta, Rm 3-524, Edmonton Clinic Health Academy, 11405 87 Avenue NW, Edmonton, Alberta T6G 1C9 Canada; 6 Department of Mathematical and Statistical Sciences, University of Alberta, Edmonton, Alberta Canada; 7 Department of Statistics and Actuarial Science, Simon Fraser University, Burnaby, British Columbia Canada

**Keywords:** Multistate models, Emergency department, Pediatrics, Asthma, Administrative data

## Abstract

**Background:**

Acute asthma is a common presentation to emergency departments (EDs) worldwide and, due to overcrowding, delays in treatment often occur. This study deconstructs the total ED length of stay into stages and estimates covariate effects on transition times for children presenting with asthma.

**Methods:**

We extracted ED presentations in 2019 made by children in Alberta, Canada for acute asthma. We used multivariable Cox regressions in a multistate model to model transition times among the stages of start, physician initial assessment (PIA), disposition decision, and ED departure.

**Results:**

Data from 6598 patients on 8270 ED presentations were extracted. The individual PIA time was longer (i.e., HR < 1) when time to the crowding metric (hourly PIA) was above 1 h (HR = 0.32; 95% CI:0.30,0.34), for tertiary (HR = 0.65; 95% CI:0.61,0.70) and urban EDs (HR = 0.77; 95% CI:0.70,0.84), for younger patients (HR = 0.99 per year; 95% CI:0.99,1.00), and for patients triaged less urgent/non-urgent (HR = 0.89; 95% CI:0.84,0.95). It was shorter for patients arriving by ambulance (HR = 1.22; 95% CI:1.04,1.42). Times from PIA to disposition decision were longer for tertiary (HR = 0.47; 95% CI:0.44,0.51) and urban (HR = 0.69; 95% CI:0.63,0.75) EDs, for patients triaged as resuscitation/emergent (HR = 0.51; 95% CI:0.48,0.54), and for patients arriving by ambulance (HR = 0.78; 95% CI:0.70,0.87). Times from disposition decision to ED departure were longer for patients who were admitted (HR = 0.16; 95% CI:0.13,0.20) or transferred (HR = 0.42; 95% CI:0.35,0.50), and for tertiary EDs (HR = 0.93; 95% CI:0.92,0.94).

**Conclusions:**

All transition times were impacted by ED presentation characteristics. The sole key patient characteristic was age and it only impacted time to PIA. ED crowding demonstrated strong effects of time to PIA but not for the transition times involving disposition decision and ED departure stages.

**Supplementary Information:**

The online version contains supplementary material available at 10.1186/s12873-021-00550-z.

## Background

Asthma is common problem and acute asthma flare-ups can be a serious condition which often requires urgent emergency department (ED) care. Though asthma can present in people of all ages, it has a higher prevalence in children [1]. Severe exacerbations require immediate treatment in the ED and often result in hospital admission. Airway interventions (e.g., intubation, chest tubes), intensive care unit (ICU) admission, and even death can occur in the most severe presentations. Fortunately most patients with acute asthma respond to treatment with bronchodilators and systemic corticosteroid agents and can be safely discharged from the ED [2].

Concerns with crowding suggest that this problem is a risk to patients with conditions like asthma, where timely care is critical. Crowding in the ED setting occurs when the demand for care cannot be met in a timely fashion. Healthcare systems worldwide face ED crowding leading to challenges in the timely delivery of healthcare to patients, patient and provider dissatisfaction, and other adverse outcomes. The problem of ED crowding is complex and is associated with input (patient volume factors), throughput (e.g., assessment and management), and output (lack of hospital beds) factors, as well as system-wide influences. Therefore, it is crucial that children presenting to the ED with asthma complications do not experience long delays prior to assessment and treatment by a physician.

Understanding ED crowding and its impacts on patients with asthma is an important step to implementing changes. In this research, we deconstruct the entire ED length of stay into different stages. This deconstruction allows for a specific analysis of the times that patients spend in the ED. The primary objective of this study is to identify factors that influence the transitions between each of these stages. The secondary objective is to determine the influence that ED crowding may have on the time spent in each stage.

## Patients and methods

### Study design

This retrospective cohort study extracted data from population-based databases of children residing in Alberta, Canada.

### Study setting and population

Alberta is a province of Canada with a population of approximately 4 million [3] and a single payer health system. The study population consisted of children aged 2 and 17 years who presented to any Alberta ED for asthma during January 1, 2019, and December 31, 2019.

### Study protocol

Data were extracted from the National Ambulatory Care Reporting System (NACRS) database [4]. Age at presentation is provided in years, sex (male/female), and mode of arrival to the ED were recorded. Triage code measures the severity of the patient’s condition according to the Canadian Triage and Acuity Scale (CTAS), where 1 = resuscitation, 2 = emergent, 3 = urgent, 4 = less urgent, and 5 = non-urgent [5, 6]. Up to ten diagnosis codes are provided using the International Statistical Classification of Diseases and Related Health Problems, Tenth Revision, Canada [7]. An asthma presentation was defined as a presentation where the primary or secondary diagnosis was for asthma (J45.x). The disposition of each patient is provided as one of 20 codes that describes their departure from the ED (e.g., discharged home, admitted to hospital) and were grouped into admitted, transferred, or discharged categories. There were 109 different EDs, two of which were pediatric. The EDs were categorized into four types: tertiary care/academic (5 EDs), urban (8), regional (5), or rural (91).

Several relevant dates and times are also available in NACRS, including registration, triage, physician initial assessment (PIA), disposition decision, and ED departure. Disposition decision time is the time that the physician decides if the patient should be admitted to hospital, discharged home, or otherwise, and ED departure time is when the patient leaves the ED. We defined the start of the ED presentation as the earliest of registration and triage time, and the end as the latest of disposition decision and ED departure time. For discharged patients, ED departure time may not be available since departure would occur once the decision was made. ED work shift (day shift 08:01–16:00, evening shift 16:01–00:00, and night shift 00:01–08:00), weekend/weekday, and season (spring = March–May, summer = June–August, fall = September–November, Winter = December–February) were determined according to the start of the ED presentation.

Data on all presentations for all ages and all conditions were also extracted from NACRS to enable calculation of an ED crowding metric. For each institution, an hourly ED crowding metric was created by calculating the median time to PIA across all patients with a presentation start time within the same hour of the same day. The hourly, ED-specific median times to PIA were further classified greater than 1 h and 1 h or less, since 1 h is the suggested benchmark median time to PIA [8]. The study data were linked to this metric through the institution and the day and hour of presentation start time.

### Key outcome measures

**Fig. 1:**
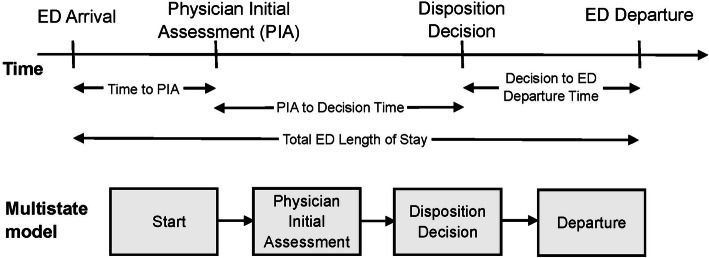
Events during an emergency department presentation, corresponding times, and a representation of the multistate model. ED = emergency department

Time to PIA was calculated as the difference between the PIA time and the start time. If the PIA time occurred prior to the start, and time to PIA was set to 0. If the PIA time was missing, the time to PIA was censored at the start time (i.e., 0). Time to disposition was calculated as the difference between the disposition decision time and the PIA time, and time to ED departure was calculated as the difference between ED departure time and the disposition decision time. If either of these times were missing, the time to event was censored at the last available time point. These four key event times characterize a patient’s flow through the ED and define four states (Fig. 1).

### Data analysis

Counts, percentages, mean, standard deviation (SD), median, and interquartile range (IQR represented as 25th percentile, 75th percentile) summarized patient demographics and ED presentation characteristics. Patients who died upon arrival to an ED were excluded as they did not contribute information regarding patient flow through the ED. Patients who left the ED prior to PIA and patients who were seen by a physician but left the ED before their disposition decision were also excluded, because there were too few for modeling. Kaplan-Meier curves summarized times in each state. We modelled the transitions from start to PIA, PIA to disposition, and disposition to ED departure using Cox proportional hazards models. Clustering by patient was used to adjust for the dependence of ED presentations made by the same patient. For each transition, age, sex, arrival mode, season, weekend indicator, shift, ED type, triage, and the crowding metric were all considered as covariates based on availability in the dataset and use in another flow paper [9]. For the disposition to ED departure transition, the disposition of the patient was also included. Initial models included one of the considered covariates at the time providing unadjusted (e.g., crude) estimates. Full models included all considered covariates. These full models were reduced by backward selection (e.g., variables excluded one at a time starting with the highest *p*-value) to include only covariates which significantly impacted state durations (*p*-value< 0.05, two-sided) to provide reduced models that are the key results presented. The proportional hazard assumptions were checked using scaled Schoefeld residuals (plots and tests) as well as plots of the log(−log (survival) versus the log of survival. CTAS was grouped into 1/2 (resuscitation/emergent), 3 (urgent), and 4/5 (less urgent/non-urgent) to meet the proportional hazards assumption. Hazard ratios (HRs) and 95% confidence intervals (CIs) were calculated. An HR > 1 indicates a higher instantaneous probability of transition to a state and consequently a faster time to transition (i.e., a shorter time in a state). All analyses were conducted in R [10] version 3.6.3 with “survival” [11, 12], “survminer” [13], and “forestplot” [14] packages.

## Results

### Demographics

There were 8320 acute asthma presentations extracted, 8270 (99.4%) of which remained for analysis after removing those who left prior to PIA or disposition or died upon arrival to the ED. The median number of ED presentations was 1 per patient (IQR 1, 1). Most of the ED presentations occurred at rural (40.2%) or tertiary (38.1%) EDs (Table 1). The majority of presentations were male (62.9%) and the mean age was 7.3 years (SD = 4.5). Presentations were mainly triaged as 1/2 (resuscitation/emergent) (38.3%) or 3 (urgent) (38.2%), while only 22.4% were triaged as 4/5 (less urgent/non-urgent). All ED types had between 35 and 42% of presentations triaged as 3 urgent. Less presentations to rural EDs were given a 1/2 (resuscitation/emergent) triage level (19.0%) compared to tertiary (56.9%) and urban (53.4%) EDs. Patients were predominantly discharged after their assessment (89.5%).

**Table 1:** Demographic and ED presentation characteristics

Variable	Count	Percentage
Total	8270	
Sex		
Male	5204	62.9
Female	3065	37.1
Age (years)		
Mean (SD^a^)	7.3	(4.5)
Season		
Winter	1737	21.0
Spring	2137	25.8
Summer	1688	20.4
Fall	1737	32.7
Weekend		
Weekday	5708	69.0
Weekend	2562	31.0
Shift		
8:01–16:00	3367	40.7
16:01–00:00	3622	43.8
00:01–8:00	1281	15.5
Arrival Mode		
Ambulance	431	5.2
No ambulance	7838	94.8
ED^b^ Type		
Rural	3325	40.2
Regional	820	9.9
Urban	974	11.8
Tertiary Care/Academic	3151	38.1
Triage Level		
1/2 – Resuscitation/Emergent	3171	38.3
3 – Urgent	3163	38.2
4/5 – Less Urgent/Non-Urgent	1851	22.4
Missing	85	1.0
PIA^c^ Metric		
> 1 h	4527	54.7
≤ 1 h	2862	34.6
Missing	881	10.7
Disposition		
Discharged	7405	89.5
Admitted	634	7.7
Transferred	231	2.8

^a^ *SD* Standard deviation; ^b^ *ED* Emergency department; ^c^ *PIA* Physician initial assessment

### State transitions

**Fig. 2:**
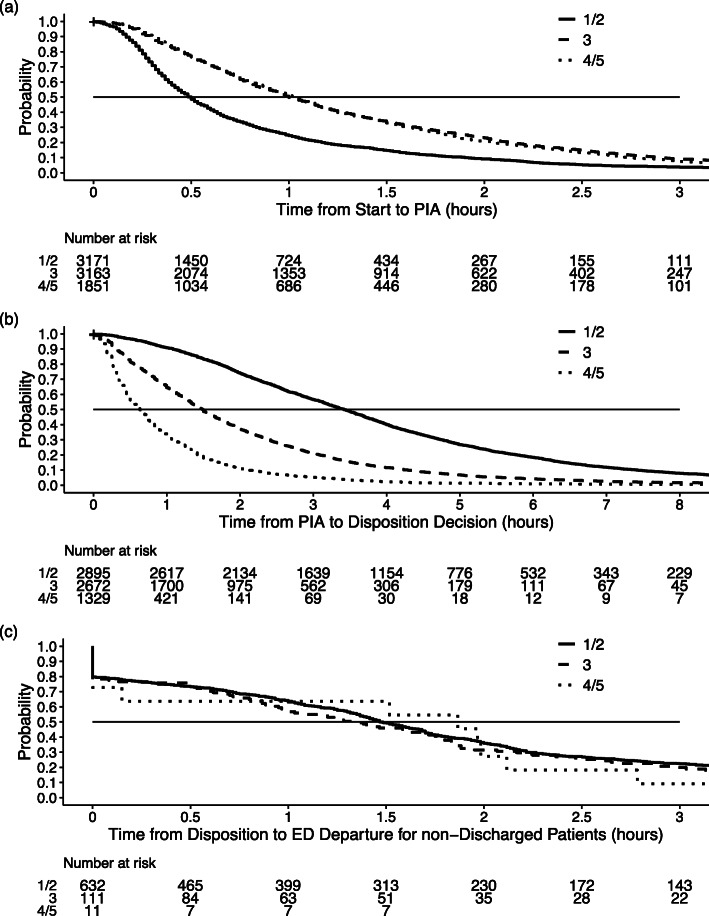
Kaplan-Meier curves by triage for (a) start to PIA, (b) PIA to disposition, and (c) disposition decision to departure. Note: Numbers < 6 suppressed for data confidentiality as per data agreement. 1/2 = resuscitation/emergent, 3 = urgent, 4/5 = less urgent/non-urgent. ED = emergency department. PIA = physician initial assessment

When examining state transitions, sample size reduction occurred due to missing time entries: 6592 presentations had available times to PIA, 8128 to disposition decision, and 4709 to ED departure. The estimated median time was 45 min (95% CI: 44 min, 47 min) from start to PIA (Table 2). Estimated times were shorter for triage 1/2 (resuscitation/emergent) (Fig. 2), arrival by ambulance, and in less crowded and less urban EDs (Supplementary Fig. 1, Additional File 1). For PIA to disposition decision, the estimated median time was 1 h53 min (95% CI: 1h50min, 1h56min). Estimated times were shorter for triage 4/5 (less urgent/non-urgent) (Fig. 2), ambulatory arrival, and in less crowded and less urban EDs (Supplementary Fig. 2, Additional File 1). The median time from disposition decision to ED departure was estimated to be 1 h46 min (95% CI: 1h39min, 1h56min) for admitted patients. The vast majority of discharged and transferred patients had the same disposition decision and ED departure times (i.e., median of 0 min).

**Table 2:** Kaplan-Meier estimated times in each state of an ED presentation, overall and by key variables

Variable	Median duration (95% CI^**a**^) (hours)		
	Start to PIA^**b**^	PIA to Disposition Decision	Disposition Decision to ED^c^ Departure (excluding discharged)
Overall	45min^d^ (44 min,47 min)	1h53min^e^ (1h50min,1h56min)	1 h28 min (1h22min,1h36min)
Sex			
Male	45 min (43 min,47 min)	1h58min (1h54min,2h2min)	1h29min (1h23min,1h41min)
Female	46 min (44 min,49 min)	1 h45 min (1h40min,1h50min)	1h24min (1h14min,1h40min)
Season			
Winter	56 min (52 min,59 min)	1 h36 min (1h30min,1h43min)	1h21min (56 min,1h40min)
Spring	48 min (45 min,50 min)	1 h39 min (1h32min,1h49min)	1 h36 min (1h25min,1h48min)
Summer	39 min (37 min,42 min)	1 h53 min (1h45min,2h2min)	1h14min (56 min,1h36min)
Fall	43 min (41 min,45 min)	2h15min (2h8min,2h22min)	1 h36 min (1h24min,1h46min)
Weekend			
Weekday	46 min (44 min,48 min)	1 h 55 min (1h51min,1h59min)	1h31min (1h22min,1h41min)
Weekend	44 min (42 min,47 min)	1 h47 min (1h40min,1h54min)	1h23min (1h14min,1h34min)
Shift			
8:01–16:00	47 min (45 min,49 min)	1 h50 min (1h44min,1h55min)	1h31min (1h21min,1h41min)
16:01–00:00	48 min (45 min,50 min)	1h49min (1h44min,1h54min)	1h22min (1h12min,1h28min)
00:01–8:00	36 min (33 min,39 min)	2h12min (2h3min,2h25min)	1 h43 min (1h30min,2h6min)
Arrival Mode			
Ambulance	28 min (25 min,32 min)	3 h43 min (3h25min,4h6min)	1 h44 min (1h35min,2h5min)
No ambulance	47 min (45 min,48 min)	1h49min (1h45min,1h52min)	1h22min (1h14min,1h29min)
Triage Level			
1/2 - Resuscitation/ Emergent	30 min (29 min,31 min)	3h25min (3h18min,3h32min)	1h28min(1h23min,1h37min)
3 - Urgent	1 h (58 min,1h3min)	1 h30 min (1h25min,1h33min)	1h21min (59 min,1h46min)
4/5 - Less Urgent/ Non-Urgent	1h2min (59 min,1h5min)	38 min (35 min,41 min)	1h52min (9 min,NA^f^)
ED Type			
Rural	40 min (37 min,41 min)	53 min (50 min,56 min)	0 min (0 min,0 min)
Tertiary Care/ Academic	50 min (48 min,54 min)	3h12min (3h5min,3h18min)	1h55min (1h46min,2h4min)
Urban	53 min (48 min,58 min)	2hmin (2h4min,2h16min)	0 min (0 min,0 min)
Regional	44 min (41 min,49 min)	1h15min (1h9min,1h21min)	1h19min (1 h,1h42min)
PIA Metric			
> 1 h	1h11min (1h9min,1h13min)	2 h10 min (2h5min,2h16min)	1h37min (1h28min,1h45min)
≤ 1 h	28 min (27 min,29 min)	1h26min (1h23min,1h30min)	1 h10 min (57 min,1h22min)

^a^ *CI* Confidence interval; ^b^ *PIA* Physician initial assessment; ^c^ *ED* Emergency department; ^d^ *min* Minute; ^e^ *h* Hour; ^f^ *NA* Not calculable due to low data counts

### Start to PIA

**Fig. 3:**
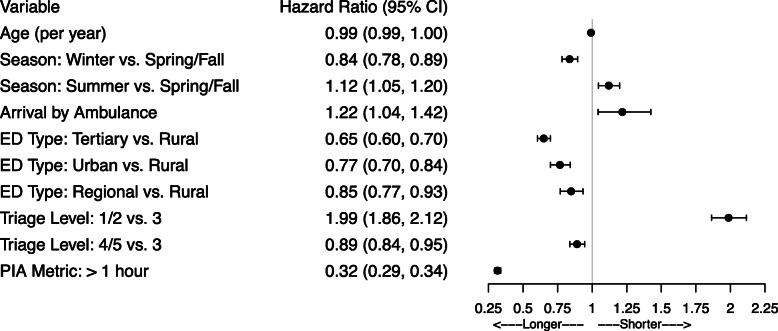
Forest plot of hazard ratios and 95% confidence intervals for the transition from start to PIA. ED = emergency department. PIA = physician initial assessment

The reduced multivariable model showed that presentations with triage level 1/2 (resuscitation/emergent) were seen faster than presentations with level 3 (urgent; HR = 1.99; 95% CI: 1.86, 2.12), and presentations arriving by ambulance were seen faster than those which were not (HR = 1.22, 95% CI: 1.04, 1.42, Fig. 3, Supplementary Table 1, Additional File 1). Presentations in the summer had a shorter time to PIA than presentations in the fall or spring. In contrast, presentations with triage level 4/5 (less urgent/non-urgent) were seen more slowly than those with level 3 (urgent), and presentations in the winter had a longer time to PIA than presentations in the fall or spring. Time to PIA was also estimated to be longest for tertiary EDs, followed by urban EDs and then regional EDs compared to rural EDs. Older patients had longer times to PIA (HR = 0.99 per year of age, 95% CI: 0.99, 1.00). Presentations starting when the hospital PIA metric was over 1 h had longer times to PIA (HR = 0.32, 95% CI: 0.29, 0.34).

### PIA to disposition decision

**Fig. 4:**
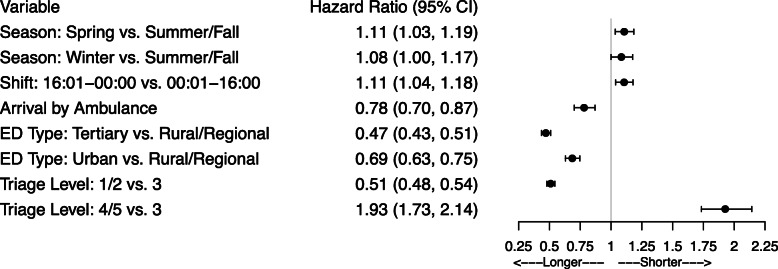
Forest plot of hazard ratios and 95% confidence intervals for the transition from PIA to disposition decision. ED = emergency department

Compared to the first transition, less covariates were found to impact the duration from PIA to disposition decision (Fig. 4; Supplementary Table 2, Additional File 1). Presentations assigned a triage level of 1/2 (resuscitation/emergent) waited longer between PIA and disposition decision than those with triage level 3 (urgent) (HR = 0.51, 95% CI: 0.48, 0.54, reduced model), and those assigned a triage level of 4/5 (less urgent/non-urgent) waited the least (HR = 1.93, 95% CI: 1.73, 2.14). Compared to the day and night shifts, presentations during the evening shift had shorter times from PIA to disposition decision. Compared to presentations in the summer or fall, times from PIA to disposition decision were shorter for presentations in the spring and winter. Times from PIA to disposition were estimated to be longer for tertiary EDs, and urban EDs than for rural and regional EDs. Time from PIA to disposition decision was also longer for those admitted by ambulance. Notably, the PIA metric did not contribute to increased times from PIA to decision.

### Disposition to departure

**Fig. 5:**
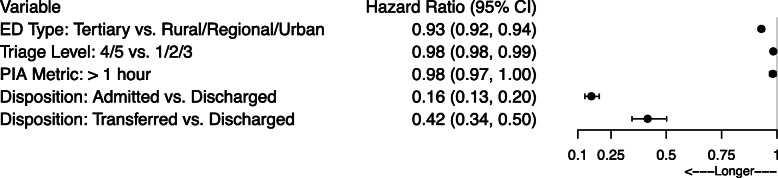
Forest plot of hazard ratios and 95% confidence intervals for the transition from disposition decision to ED departure. ED = emergency department. PIA = physician initial assessment

Times from disposition decision to ED departure were predominantly impacted by admission/transfer or discharge disposition decision (Fig. 5; Supplementary Table 3, Additional File 1, reduced model). There were longer times from disposition decision to ED departure for both admitted (HR = 0.16, 95% CI: 0.13, 0.20) and transferred (HR = 0.42, 95% CI: 0.35, 0.50) patients than for discharged patients. Patient presentations assigned a triage code of 4/5 (less urgent/non-urgent) took longer to depart the ED after their disposition decision than presentations assigned to other triage levels. Compared to the other ED types, patients took longer to depart from tertiary EDs after their disposition decision.

## Discussion

In this population-based study of ED visits for acute asthma involving over 8000 children, the length of stay was divided into transitions among PIA, disposition decision, and ED departure to assess the transition-specific effects of covariates. Important findings at each transition stage have implications for planners. Times to PIA were longer for children who were older and children who presented during winter, to non-rural EDs, with lower acuity, and when EDs were crowded. Times from PIA to disposition decision were longer for children who arrived by ambulance, presented to tertiary and urban EDs, and had higher acuity. Children who were admitted or transferred had longer times from disposition decision to ED departure.

Why are these finding important? It is now well accepted that systemic corticosteroids are the cornerstone of treatment for acute asthma in children and adults [2]. Moreover, intravenous and oral systemic corticosteroids are similarly effective for treating exacerbations severe enough to require ED care; most EDs opt for oral delivery except in the most severe cases [15, 16]. Finally, the earlier these agents can be delivered, the more effective they are at preventing hospitalization [15]. Since delays in delivery of systemic corticosteroid agents in EDs have been observed [16], many pediatric EDs have implemented strategies to deliver these agents as soon as possible after presentations for asthma [17].

These results require further analysis to provide nuances that are required for implementing changes. The effects of acuity aligned with expectations, since patients with more urgent conditions were seen faster by a physician, yet took longer to reach a disposition decision, likely because presentations of severe asthma require more aggressive treatment and prolonged periods of observation. While we did not see a difference in times from disposition decision to ED departure between triage code resuscitation/emergent and triage code urgent, the impact was likely captured through the disposition; patients requiring more urgent care were more frequently admitted to hospital. Emergency type impacted all transitions, with tertiary EDs experiencing longer transition times in all cases, and urban EDs experiencing longer transition times between start to PIA and PIA to disposition decision. Previous literature has suggested that patients visiting more urban EDs spend longer in the ED, partly due to these EDs experiencing higher volumes, more complex cases, and a lower proportion of less-urgent cases than other EDs; median time to PIA for urgent presentations in 2003–2004 (primarily calculated from Ontario-based EDs) was found to be approximately 25 min for low-volume community EDs and 1 h10 min for high-volume community EDs [18]. Given that the tertiary and urban EDs included in our study did have smaller proportions of less urgent/non-urgent presentations than the rural EDs, these previous results are in agreement with our own findings. Sex was not found to impact times among any of the transitions, contrasting the Ohio study, which suggested that males are seen by a physician slightly faster than females [9]. Our results were consistent with the Ohio study in that time to PIA was shortest in the summer and slowest in the winter [9]. Also of note are the relatively few predictors that influence the time from PIA to disposition decision. These results suggest that once patients are seen, management decisions are more uniform.

The impact of ED crowding was clear in the transition from arrival to PIA. Patients arriving when the average PIA time was longer also had longer times to PIA. This finding demonstrates the importance both of adjusting for the crowding metric when estimating effects of other covariates and of reducing ED crowding to allow for faster time to PIA. The crowding metric was, however, not significantly associated with the transition from PIA to disposition decision and had only a minimal effect on time from disposition decision to ED departure. This result is contrary to a study which found that part of the total ED length of stay for asthma patients could be attributed to the effect of ED crowding on time to medication order [19]. Our findings suggest that once seen by a physician, ED crowding does not impact the management decisions for patients presenting with asthma and ED efforts to address Crowding should focus on shortening the PIA time.

Crowding is a complex, multi-factorial problem and efforts to address it are important healthcare interventions. Clearly, crowding in EDs was demonstrated in this sample of high-volume and diverse EDs in one Canadian province. While the factors identified to impact each transition time may not all be modifiable the results indicate where time-reduction efforts should focus. The longer times to PIA and disposition decision experienced by tertiary and urban EDs and the times that admitted patients spend waiting for an in-patient bed are of particular concern.

This study used population-based data to create a comprehensive sample of children with an important condition requiring timely care. While multistate models have been utilized in health research to model events such as repeated hospitalizations [20–22] or symptom progression [23, 24], to our knowledge, only one other study has implemented multistate models to analyze transition times among multiple stages of an ED presentation. That study analyzed the flow of pediatric patients through the ED at Nationwide Children’s Hospital, Ohio, USA, and included patients of any diagnosis [9]. Our study is comparable but differs in scope, as we include all Alberta EDs, focus on mixed and pediatric EDs, and consider only patients presenting with asthma.

From the perspective of ED administrators and clinicians, the implications of this research are that bottlenecks for assessing pediatric patients with asthma are important to identify. Since EDs and hospitals are complex and unique organizations, a one-size-fits all solution is unlikely to be effective. In addition, since there is evidence that expediting the administration of anti-inflammatory agents can reduce ED LOS and hospitalization [15], pediatric EDs should implement strategies to expedite and increase the delivery of systemic corticosteroid agents (e.g., care pathways, administration by non-physician providers, etc.) [17].

Our study has limitations. We focused on the times of each state transition; we acknowledge that there could be errors in the reporting of times and some times were missing for PIA and disposition decision. The missing times largely occurred in rural EDs, so there is differential censoring by ED type. Another limitation was our inability to model those who left without being seen or left against medical advice in a meaningful way; however, these were infrequent (< 1%). Our study could have also been improved if we were able to account for other features of the ED facility staffing, such as the number of physicians working in the ED during an ED presentation. Such data are not included in NACRS and we did include the PIA metric as a proxy for crowding that has been shown to increase ED length of stay in children presenting with asthma [25].

## Conclusions

In conclusion, patients with acute asthma in Alberta EDs experience delays in assessment, management, and disputation decisions, especially in high volume EDs. Times to PIA are longer for older children presenting with acute asthma who have lower acuity and do not arrive by ambulance, present during the winter, present at tertiary and urban EDs, and present to EDs when the PIA metric is more than 1 h. Patients with asthma experience delays in receiving a disposition decision if the acuity of their presentation is more severe, they arrive by ambulance, and present at a tertiary or urban ED. These results suggest that overcrowding has an impact on acute asthma care and indicates interventions should be considered, implemented and evaluated to address the bottlenecks in care, especially in tertiary and urban EDs.

## Supplementary Information

**Additional file 1. **Supplementary Tables. Hazard ratios, 95% confidence intervals, and *p*-values for different models. Supplementary Figures. Kaplan-Meier curves for different transitions and characterisitcs.

## Data Availability

Data is the property of Alberta Health and the authors are not allowed to provide the data. Requests can be made for the same data from Alberta Health for researchers who meet the criteria for access to confidential data. Researchers are welcome to inquire for further information at Health.RESDATA@gov.ab.ca.

## Abbreviations

CI: Confidence interval
CTAS: Canadian triage and acuity scale
ED: Emergency department
h: Hour
HR: Hazard ratio
min: Minute
NACRS: National ambulatory care reporting system
PIA: Physician initial assessment
