# Supplementary material for: Evaluating the transitions in care for children presenting with acute asthma to emergency departments: a retrospective cohort study
Source: BMC Emerg Med. 2021 Dec 7;21:153. doi: 10.1186/s12873-021-00550-z (PMC8650289; doi:10.1186/s12873-021-00550-z)
Supplement: Supplementary file 1 — Additional file 1. Supplementary Tables. Hazard ratios, 95% confidence intervals, and p-values for different models. Supplementary Figures. Kaplan-Meier curves for different transitions and characterisitcs. [file 12873_2021_550_MOESM1_ESM.docx]

**Additional File 1**

**Evaluating the transitions in care for children presenting with acute asthma to Emergency Departments: a retrospective cohort study**

Kimberly R. Kroetch^1^_,_ Brian H. Rowe^2,3,4^_,_ Rhonda J. Rosychuk^5,6,7^

**Affiliations:**

^1^Department of Mathematics and Statistics, Faculty of Arts and Science, MacEwan University, Edmonton, Alberta, Canada T5J 4S2

^2^Department of Emergency Medicine, Faculty of Medicine & Dentistry, University of Alberta, Edmonton, Alberta, Canada T6G 2R7

^3^School of Public Health, University of Alberta, Edmonton, Alberta, Canada

^4^Department of Medicine, Faculty of Medicine & Dentistry, University of Alberta, Edmonton, Alberta, Canada

^5^Department of Pediatrics, Faculty of Medicine & Dentistry, University of Alberta, Edmonton, Alberta, Canada T6G 1C9

^6^Department of Mathematical and Statistical Sciences, University of Alberta, Edmonton, Alberta, Canada

^7^Department of Statistics and Actuarial Science, Simon Fraser University, Burnaby, British Columbia, Canada

Supplementary Table 1. Unadjusted and adjusted hazard ratios (HRs) for univariable and multivariable models of flow from start to physician initial assessment (PIA).

|  | **Univariable Models** | | | **Multivariable Models** | | | | | |
| --- | --- | --- | --- | --- | --- | --- | --- | --- | --- |
|  |  |  |  | **Full Model** | | | **Reduced Model** | | |
| **Variables** | **HR**^a^ | **(95% CI**^b^**)** | **p-value** | **HR** | **(95% CI)** | **p-value** | **HR** | **(95% CI)** | **p-value** |
| Sex |  |  |  |  |  |  |  |  |  |
| Male | Reference | | | Reference | | |  |  |  |
| Female | 0.97 | (0.93, 1.02) | 0.32 | 0.99 | (0.94, 1.05) | 0.74 |  |  |  |
| Age (per year) | 0.99 | (0.99, 1.00) | 0.03 | 0.99 | (0.99, 1.00) | 0.01 | 0.99 | (0.99, 1.00) | 0.01 |
| Season |  |  |  |  |  |  |  |  |  |
| Spring | 0.95 | (0.89, 1.01) | 0.09 | 1.04 | (0.98, 1.11) | 0.19 | Reference^c^ | | |
| Summer | 1.13 | (1.05, 1.21) | <0.01 | 1.14 | (1.06, 1.23) | <0.01 | 1.12 | (1.05, 1.20) | <0.01 |
| Fall | Reference | | | Reference | | | Reference^c^ | | |
| Winter | 0.80 | (0.75, 0.85) | <0.01 | 0.85 | (0.79, 0.92) | <0.01 | 0.84 | (0.78, 0.89) | <0.01 |
| Weekend | 1.08 | (1.03, 1.13) | <0.01 | 1.03 | (0.98, 1.09) | 0.25 |  |  |  |
| Shift |  |  |  |  |  |  |  |  |  |
| 08:01-16:00 | Reference | | | Reference | | |  |  |  |
| 16:01-00:00 | 0.95 | (0.90, 1.00) | 0.04 | 0.99 | (0.94, 1.04) | 0.67 |  |  |  |
| 00:01-08:00 | 1.16 | (1.07, 1.25) | <0.01 | 1.03 | (0.95, 1.13) | 0.47 |  |  |  |
| Arrival by Ambulance | 1.27 | (1.10, 1.45) | <0.01 | 1.21 | (1.04, 1.42) | 0.02 | 1.22 | (1.04, 1.42) | 0.01 |
| Emergency Department Type |  |  |  |  |  |  |  |  |  |
| Rural | Reference | | | Reference | | | Reference | | |
| Regional | 0.89 | (0.82, 0.96) | <0.01 | 0.84 | (0.76, 0.93) | <0.01 | 0.85 | (0.77, 0.93) | <0.01 |
| Urban | 0.76 | (0.71, 0.82) | <0.01 | 0.76 | (0.69, 0.84) | <0.01 | 0.77 | (0.70, 0.84) | <0.01 |
| Tertiary Care/Academic | 0.68 | (0.64, 0.72) | <0.01 | 0.65 | (0.60, 0.70) | <0.01 | 0.65 | (0.60, 0.70) | <0.01 |
| Triage Level |  |  |  |  |  |  |  |  |  |
| 1/2 Resuscitation/Emergent | 1.74 | (1.64, 1.85) | <0.01 | 1.99 | (1.87, 2.12) | <0.01 | 1.99 | (1.86, 2.12) | <0.01 |
| 3 Urgent | Reference | | | Reference | | | Reference | | |
| 4/5 Less Urgent/Non-Urgent | 1.03 | (0.98, 1.09) | 0.26 | 0.89 | (0.84, 0.95) | <0.01 | 0.89 | (0.84, 0.95) | <0.01 |
| PIA Metric |  |  |  |  |  |  |  |  |  |
| ≤ 1 hour | Reference | | | Reference | | | Reference | | |
| > 1 hour | 0.31 | (0.29, 0.33) | <0.01 | 0.32 | (0.30, 0.34) | <0.01 | 0.32 | (0.29, 0.34) | <0.01 |

^a^ HR=hazard ratio; ^b^ CI=confidence interval; ^c^ Spring and Fall are collapsed to become the reference group in the reduced model

Supplementary Table 2. Unadjusted and adjusted hazard ratios (HRs) for univariable and multivariable models of flow from physician initial assessment to disposition decision.

|  | **Univariable Models** | | | **Multivariable Models** | | | | | |
| --- | --- | --- | --- | --- | --- | --- | --- | --- | --- |
|  |  |  |  | **Full Model** | | | **Reduced Model** | | |
| **Variables** | **HR**^a^ | **(95% CI**^b^**)** | **p-value** | **HR** | **(95% CI)** | **p-value** | **HR** | **(95% CI)** | **p-value** |
| Sex |  |  |  |  |  |  |  |  |  |
| Male | Reference | | | Reference | | |  |  |  |
| Female | 1.03 | (0.97, 1.08) | 0.32 | 1.02 | (0.95, 1.09) | 0.55 |  |  |  |
| Age (per year) | 1.05 | (1.04, 1.05) | <0.01 | 1.01 | (1.00, 1.02) | 0.10 |  |  |  |
| Season |  |  |  |  |  |  |  |  |  |
| Spring | 1.23 | (1.16, 1.31) | <0.01 | 1.13 | (1.05, 1.22) | <0.01 | 1.11 | (1.03, 1.19) | <0.01 |
| Summer | 1.13 | (1.06, 1.20) | <0.01 | 1.04 | (0.97, 1.13) | 0.29 | Reference^c^ | | |
| Fall | Reference | | | Reference | | |  |  |  |
| Winter | 1.24 | (1.15, 1.33) | <0.01 | 1.11 | (1.02, 1.21) | 0.02 | 1.08 | (1.00, 1.17) | 0.05 |
| Weekend | 1.07 | (1.02, 1.13) | 0.01 | 1.06 | (1.00, 1.13) | 0.07 |  |  |  |
| Shift |  |  |  |  |  |  |  |  |  |
| 08:01-16:00 | Reference | | | Reference | | | Reference^d^ | | |
| 16:01-00:00 | 1.02 | (0.97, 1.07) | 0.49 | 1.08 | (1.01, 1.16) | 0.03 | 1.11 | (1.04, 1.18) | <0.01 |
| 00:01-08:00 | 0.85 | (0.79, 0.90) | <0.01 | 0.93 | (0.86, 1.01) | 0.07 | Reference^d^ | | |
| Arrival by Ambulance | 0.57 | (0.53, 0.62) | <0.01 | 0.79 | (0.70, 0.88) | <0.01 | 0.78 | (0.70, 0.87) | <0.01 |
| Emergency Department Type |  |  |  |  |  |  |  |  |  |
| Rural | Reference | | | Reference | | | Reference^e^ | | |
| Regional | 0.89 | (0.79, 0.99) | 0.03 | 0.93 | (0.82, 1.06) | 0.27 |  |  |  |
| Urban | 0.52 | (0.47, 0.57) | <0.01 | 0.68 | (0.62, 0.75) | <0.01 | 0.69 | (0.63, 0.75) | <0.01 |
| Tertiary Care/Academic | 0.36 | (0.33, 0.39) | <0.01 | 0.47 | (0.43, 0.52) | <0.01 | 0.47 | (0.43, 0.51) | <0.01 |
| Triage Level |  |  |  |  |  |  |  |  |  |
| 1/2 Resuscitation/Emergent | 0.47 | (0.44, 0.49) | <0.01 | 0.52 | (0.48, 0.55) | <0.01 | 0.51 | (0.48, 0.54) | <0.01 |
| 3 Urgent | Reference | | | Reference | | | Reference | | |
| 4/5 Less Urgent/Non-Urgent | 2.27 | (2.05, 2.51) | <0.01 | 1.90 | (1.71, 2.11) | <0.01 | 1.93 | (1.73, 2.14) | <0.01 |
| PIA Metric |  |  |  |  |  |  |  |  |  |
| ≤ 1 hour | Reference | | | Reference | | |  |  |  |
| > 1 hour | 0.78 | (0.75, 0.83) | <0.01 | 0.97 | (0.91, 1.04) | 0.44 |  |  |  |

^a^ HR=hazard ratio; ^b^ CI=confidence interval; ^c^ Summer and Fall are collapsed to become the reference group in the reduced model; ^d^ 08:01-16:00 and 00:01-08:00 are collapsed to become the reference group in the reduced model; ^e^ Rural and Regional are collapsed to become the reference group in the reduced model

Supplementary Table 3. Unadjusted and adjusted hazard ratios (HRs) for univariable and multivariable models of flow from disposition to departure.

|  | **Univariable Models** | | | **Multivariable Models** | | | | | |
| --- | --- | --- | --- | --- | --- | --- | --- | --- | --- |
|  |  |  |  | **Full Model** | | | **Reduced Model** | | |
| **Variables** | **HR**^a^ | **(95% CI**^b^**)** | **p-value** | **HR** | **(95% CI)** | **p-value** | **HR** | **(95% CI)** | **p-value** |
| Sex |  |  |  |  |  |  |  |  |  |
| Male |  | Reference |  |  | Reference |  |  |  |  |
| Female | 1.00 | (0.98, 1.02) | 0.99 | 1.00 | (0.98, 1.02) | 0.78 |  |  |  |
| Age (per year) | 1.01 | (1.00, 1.01) | <0.01 | 1.00 | (1.00, 1.00) | 0.19 |  |  |  |
| Season |  |  |  |  |  |  |  |  |  |
| Spring | 1.04 | (1.01, 1.06) | <0.01 | 1.01 | (0.99, 1.03) | 0.36 |  |  |  |
| Summer | 1.05 | (1.02, 1.07) | <0.01 | 1.02 | (1.00, 1.04) | 0.04 |  |  |  |
| Fall | Reference | | | Reference | | |  |  |  |
| Winter | 1.05 | (1.02, 1.08) | <0.01 | 1.02 | (0.99, 1.04) | 0.22 |  |  |  |
| Weekend | 1.02 | (1.00, 1.04) | 0.11 | 1.01 | (0.99, 1.03) | 0.21 |  |  |  |
| Shift |  |  |  |  |  |  |  |  |  |
| 08:01-16:00 | Reference | | | Reference | | |  |  |  |
| 16:01-00:00 | 0.99 | (0.97, 1.01) | 0.46 | 0.99 | (0.97, 1.01) | 0.40 |  |  |  |
| 00:01-08:00 | 0.96 | (0.94, 0.99) | 0.01 | 1.00 | (0.98, 1.02) | 0.82 |  |  |  |
| Arrival by Ambulance | 0.64 | (0.59, 0.69) | <0.01 | 0.87 | (0.79, 0.96) | 0.01 |  |  |  |
| Emergency Department Type |  |  |  |  |  |  |  |  |  |
| Rural | Reference | | | Reference | | | Reference^c^ | | |
| Regional | 0.93 | (0.91, 0.96) | <0.01 | 1.00 | (0.97, 1.02) | 0.87 |  |  |  |
| Urban | 0.99 | (0.98, 1.00) | 0.05 | 1.00 | (0.98, 1.02) | 0.92 |  |  |  |
| Tertiary Care/Academic | 0.83 | (0.82, 0.85) | <0.01 | 0.93 | (0.92, 0.95) | <0.01 | 0.93 | (0.92, 0.94) | <0.01 |
| Triage Level |  |  |  |  |  |  |  |  |  |
| 1/2 Resuscitation/Emergent | 0.86 | (0.84, 0.88) | <0.01 | 1.01 | (0.99, 1.03) | 0.31 | Reference^d^ | | |
| 3 Urgent | Reference | | | Reference | | |  |  |  |
| 4/5 Less Urgent/Non-Urgent | 1.03 | (1.02, 1.04) | <0.01 | 0.99 | (0.98, 1.00) | 0.03 | 0.98 | (0.98, 0.99) | <0.01 |
| PIA Metric |  |  |  |  |  |  |  |  |  |
| ≤ 1 hour | Reference | | | Reference | | | Reference | | |
| > 1 hour | 0.94 | (0.93, 0.96) | <0.01 | 0.98 | (0.97, 1.00) | 0.02 | 0.98 | (0.97, 1.00) | 0.01 |
| Disposition |  |  |  |  |  |  |  |  |  |
| Discharged | Reference | | | Reference | | | Reference | | |
| Admitted | 0.16 | (0.14, 0.20) | <0.01 | 0.16 | (0.13, 0.20) | <0.01 | 0.16 | (0.13, 0.20) | <0.01 |
| Transferred | 0.42 | (0.35, 0.51) | <0.01 | 0.42 | (0.35, 0.50) | <0.01 | 0.42 | (0.34, 0.50) | <0.01 |

^a^ HR=hazard ratio; ^b^ CI=confidence interval; ^c^ Rural, Regional, and Urban are collapsed to become the reference group in the reduced model; ^d^ Triage levels 1, 2, and 3 are collapsed to become the reference group in the reduced model

Supplementary Figure 1. Kaplan-Meier curves according to arrival by ambulance, emergency department (ED) type, and physician initial assessment (PIA) metric for patient transition from start to PIA.

**
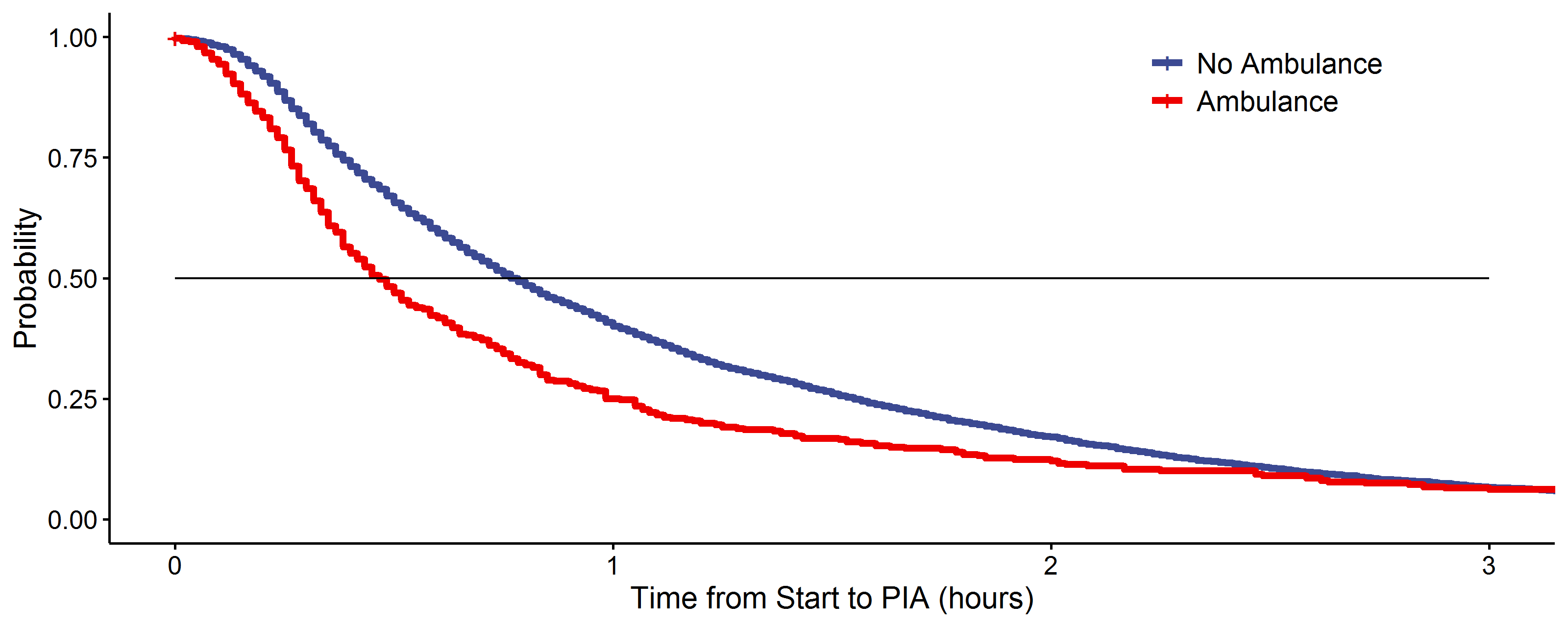
**


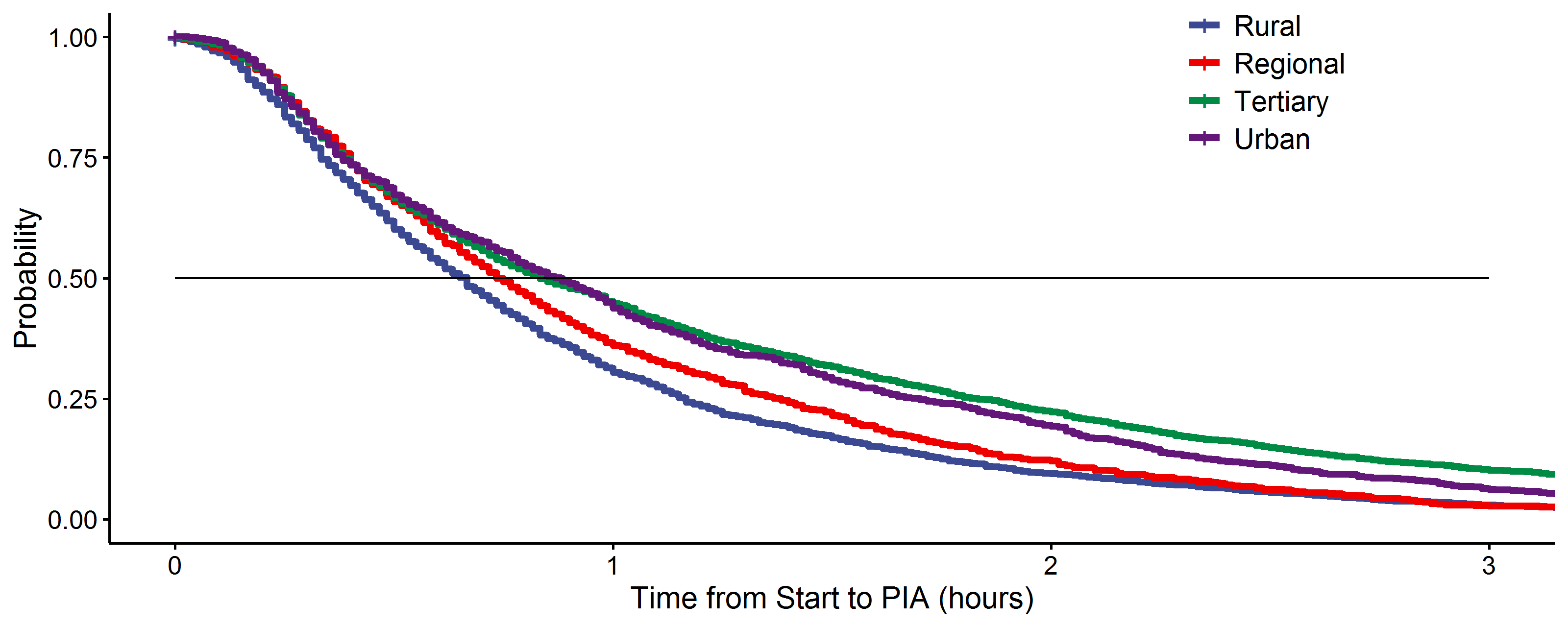

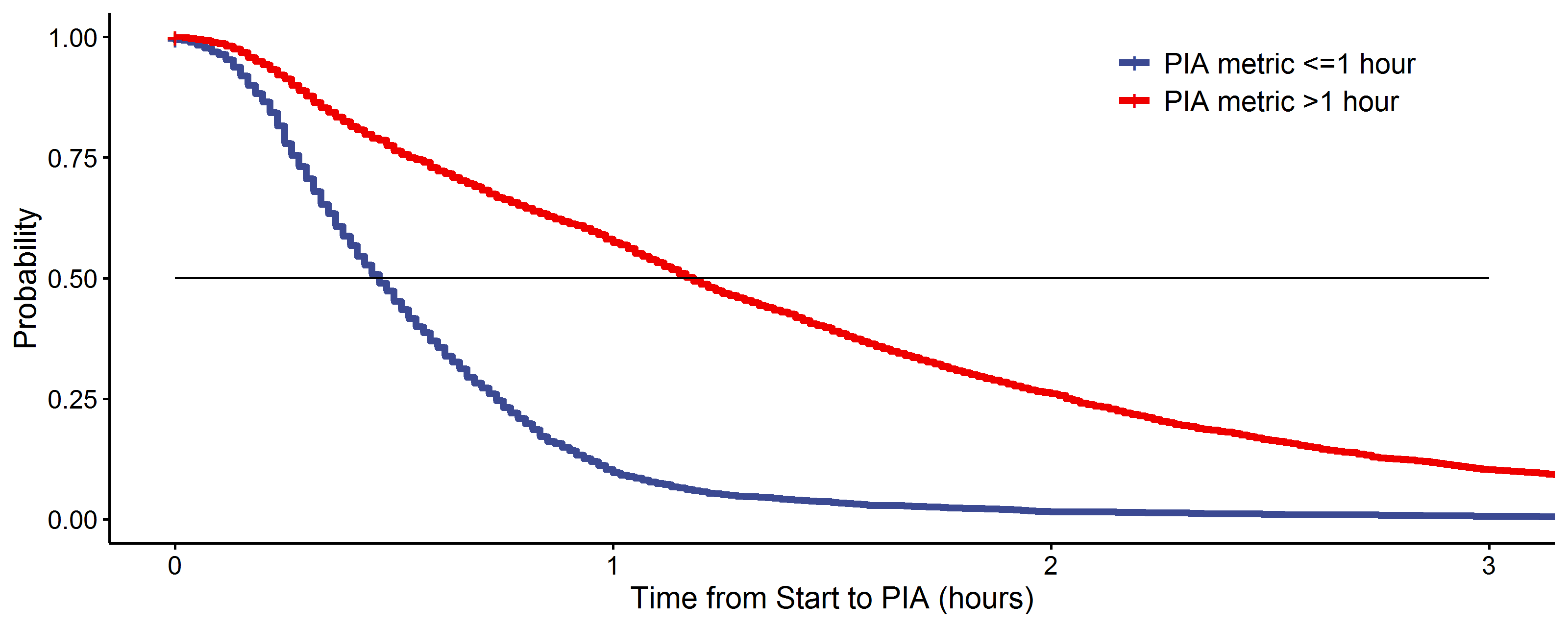


Note: horizontal line indicates the median times of the groups.

Supplementary Figure 2. Kaplan-Meier curves according to arrival by ambulance, emergency department (ED) type, and physician initial assessment (PIA) metric for patient transition from PIA to disposition decision.


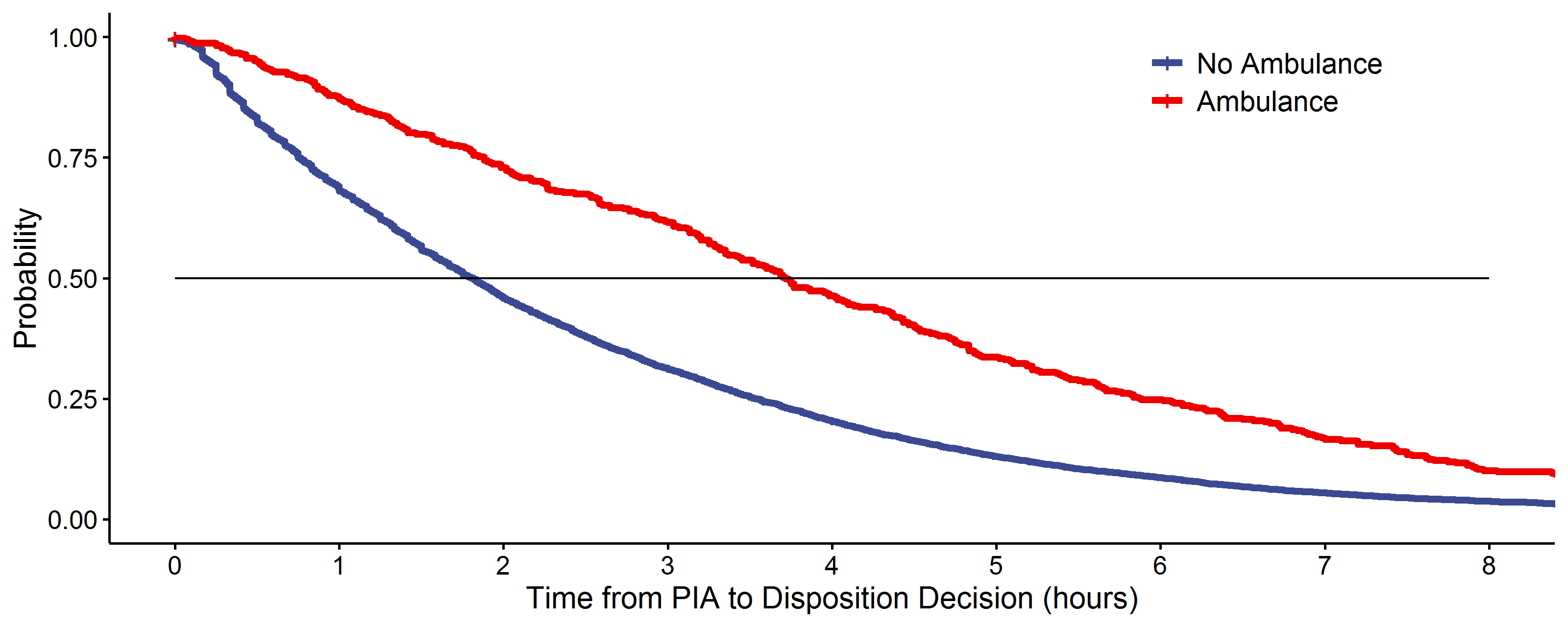

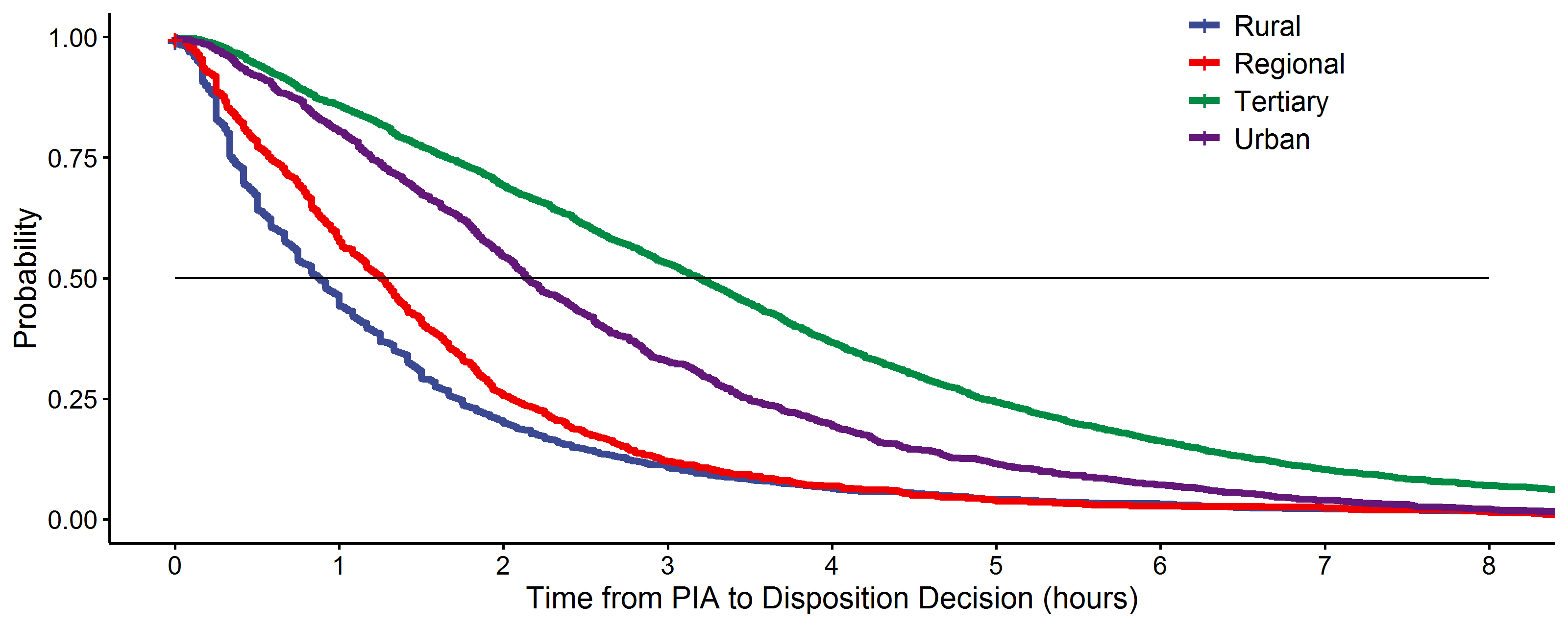

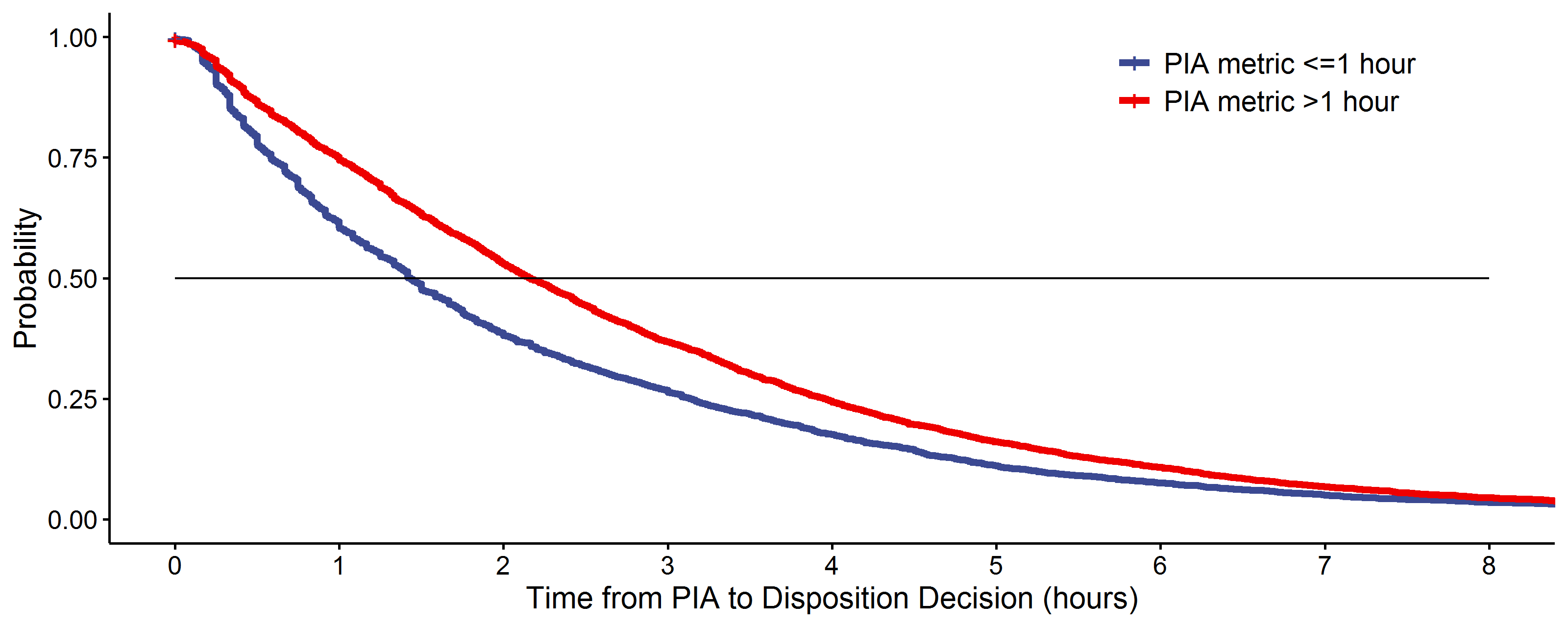


Note: horizontal line indicates the median times of the groups.
